# Saliency-driven explainable deep learning in medical imaging: bridging visual explainability and statistical quantitative analysis

**DOI:** 10.1186/s13040-024-00370-4

**Published:** 2024-06-22

**Authors:** Yusuf Brima, Marcellin Atemkeng

**Affiliations:** 1 Computer Vision, Institute of Cognitive Science, Osnabrück University, Osnabrueck, D-49090 Lower Saxony Germany; 2 Department of Mathematics, Rhodes University, Grahamstown, 6140 Eastern Cape South Africa

## Abstract

Deep learning shows great promise for medical image analysis but often lacks explainability, hindering its adoption in healthcare. Attribution techniques that explain model reasoning can potentially increase trust in deep learning among clinical stakeholders. In the literature, much of the research on attribution in medical imaging focuses on visual inspection rather than statistical quantitative analysis.

In this paper, we proposed an image-based saliency framework to enhance the explainability of deep learning models in medical image analysis. We use adaptive path-based gradient integration, gradient-free techniques, and class activation mapping along with its derivatives to attribute predictions from brain tumor MRI and COVID-19 chest X-ray datasets made by recent deep convolutional neural network models.

The proposed framework integrates qualitative and statistical quantitative assessments, employing Accuracy Information Curves (AICs) and Softmax Information Curves (SICs) to measure the effectiveness of saliency methods in retaining critical image information and their correlation with model predictions. Visual inspections indicate that methods such as ScoreCAM, XRAI, GradCAM, and GradCAM++ consistently produce focused and clinically interpretable attribution maps. These methods highlighted possible biomarkers, exposed model biases, and offered insights into the links between input features and predictions, demonstrating their ability to elucidate model reasoning on these datasets. Empirical evaluations reveal that ScoreCAM and XRAI are particularly effective in retaining relevant image regions, as reflected in their higher AUC values. However, SICs highlight variability, with instances of random saliency masks outperforming established methods, emphasizing the need for combining visual and empirical metrics for a comprehensive evaluation.

The results underscore the importance of selecting appropriate saliency methods for specific medical imaging tasks and suggest that combining qualitative and quantitative approaches can enhance the transparency, trustworthiness, and clinical adoption of deep learning models in healthcare. This study advances model explainability to increase trust in deep learning among healthcare stakeholders by revealing the rationale behind predictions. Future research should refine empirical metrics for stability and reliability, include more diverse imaging modalities, and focus on improving model explainability to support clinical decision-making.

## Background

The field of medical image analysis has seen significant advancements in explainability methods for deep learning (DL) models, driven by the imperative for *trustworthy* artificial intelligence systems in healthcare [1]. Traditional medical imaging modalities like Computed Tomography (CT), Magnetic Resonance Imaging (MRI), Functional Magnetic Resonance Imaging (fMRI), Positron Emission Tomography (PET), Mammography, Ultrasound, and X-ray play a crucial role in disease detection and diagnosis, often relying on the expertise of radiologists and physicians [2]. However, the healthcare field faces a growing demand for skilled professionals, leading to potential fatigue and highlighting the need for *computer-aided diagnostic* (CAD) tools. The rapid advancements in DL architectures and compute have fueled significant progress in automated medical image analysis [3–7]. The maturation of DL offers a promising solution, accelerating the adoption of computer-assisted systems to support experts and reduce reliance on manual analysis. DL holds particular promise for democratizing healthcare globally by alleviating the cost burden associated with scarce expertise [8]. However, successful clinical adoption hinges on establishing trust in the *robustness* and *explainability* of these models [9]. Despite their inherent complexity, DL models can be illuminated to understand their inference mechanisms, that is, how they process medical images to generate *predictions*. An adjacent line of work, *explainability*, focuses on understanding the inner workings of the models, while *explainability* focuses on explaining the decisions made by these models. Explainable models enable a human-in-the-loop approach, enhancing diagnostic performance through collaboration between domain experts and artificial intelligence.

Various techniques have been proposed, each with distinct advantages and limitations. Concept learning, for example, facilitates multi-stage prediction by leveraging high-level concepts. Studies such as [10–12] illustrate the potential of concept learning in disease categorization. However, these methods often require extensive annotation to define concepts accurately and risk information leakage if concepts do not align well with the disease pathology. Case-Based Models (CBMs) learn class-specific, disentangled representations and feature mappings, achieving final classification through similarity measurements between input images and stored base templates [13–15]. While CBMs are robust to noise and compression artifacts, their training is complex, particularly for the large and diverse datasets typical of medical imaging. Counterfactual explanation methods generate pseudo-realistic perturbations of input images to produce opposite predictions, aiming to identify influential features for the model’s original prediction. However, generating realistic perturbations for medical images, which often contain subtle anatomical details, is challenging and can lead to misleading explanations [16–23]. Unrealistic perturbations compromise the trustworthiness of these explanations. Another approach involves visualizing internal network representations of learned features in CNN kernels [24]. Interpreting these feature maps in the context of medical image analysis is difficult due to the abstract nature of the features learned by DL models [25, 26]. This abstraction challenges human experts in deriving clinically meaningful insights.

Attribution maps are visual representations that highlight regions of an image most relevant to the predictions made by a DL model. Serving as potent post-hoc explainability tools, these maps provide crucial insights into how models make decisions based on input images. Several studies have demonstrated the application of attribution maps in medical imaging tasks. For instance, Bohle et al. [27] utilized layer-wise relevance propagation to elucidate deep neural network decisions in MRI-based Alzheimer’s disease classification. Camalan et al. [28] employed a deep CNN-based Grad-CAM approach for classifying oral lesions in clinical photographs. Similarly, Kermany et al. [29] applied Grad-CAM for oral dysplasia classification. Shi et al. presented an explainable attention-based model for COVID-19 automatic diagnosis, showcasing the integration of attention mechanisms to improve explainability in radiographic imaging [30]. Another study by Shi et al. introduced an attention transfer deep neural network for COVID-19 automatic diagnosis, further enhancing the explainability and performance of diagnostic models [31]. Recently, Nhlapho et al. [32] presented an overview of select image-based attribution methods for brain tumor detection, though their approach lacked ground-truth segmentation masks and did not quantitatively evaluate the chosen saliency methods.

Building on these efforts, our research leverages both gradient-based and gradient-free image-based saliency methods. However, the deployment of attribution maps alone is insufficient for establishing comprehensive model explainability. A rigorous evaluation framework is essential. We propose a comprehensive evaluation framework that extends beyond qualitative assessment. This framework includes metrics specifically designed to evaluate image-based saliency methods. By incorporating performance information curves (PICs) such as Accuracy Information Curves (AICs) and Softmax Information Curves (SICs), we objectively assess the correlation between saliency map intensity and model predictions. This robust evaluation aims to enhance the transparency and trustworthiness of DL models in clinical settings. Given this context, this paper centers on *How effective are state-of-the-art (SoTA) image-based saliency methods in aiding the explainability of DL models for medical image analysis tasks?* By investigating this question, we aim to contribute to the broader effort of enhancing the trustworthiness, transparency, and reliability of DL applications in healthcare.

To this end, we leverage the proposed framework to systematically analyze model predictions on brain tumor MRI [33] and COVID-19 chest X-ray [34] datasets. Resulting attribution maps highlight the salient features within the input images that most significantly influence the model’s predictions. By evaluating these techniques both qualitatively and quantitatively across different SoTA DL architectures and the aforementioned medical imaging modalities, we aim to assess their effectiveness in promoting explainability. Our assessment is focused on several key aspects:

- *Clarity of Insights:* Do these saliency methods provide clear non-spurious and explainable insights into the relationship between medical image features and model predictions? We achieve this assessment by comparing the highlighted features in the attribution maps with the known anatomical structures and disease signatures relevant to the specific medical imaging task (e.g., brain tumor location in MRI).
- *Biomarker Identification:* Can these techniques aid in identifying potential biomarkers for disease detection or classification? We investigate whether the saliency methods consistently highlight specific image features that correlate with known or emerging disease biomarkers. This analysis can provide valuable insights into potential new avenues for clinical research.
- *Model Bias Detection:* Do saliency methods help uncover potential biases within the DL used for medical image analysis? We explore whether the saliency maps reveal a consistent focus on irrelevant features or artifacts that might not be clinically meaningful. This analysis can help identify potential biases in the training data or model architecture that may require mitigation strategies.
- *Quantitative Effectiveness:* How quantitatively effective are these methods in capturing the relationship between image features and model predictions? We explore this by employing PICs such as AICs and SICs. These metrics assess the correlation between the saliency map intensity and the model’s accuracy or class probabilities.

### Contributions

We proposed a comprehensive framework to evaluate SoTA image-based saliency methods applied to Deep Convolutional Neural Networks (CNNs) for medical image classification tasks. Our study included MRI and X-ray modalities, focusing on tasks such as brain tumor classification and COVID-19 detection within these respective imaging techniques. For a novel quantitative evaluation, beyond the visual inspection of saliency maps, we used AICs and SICs to measure the effectiveness of the saliency methods. AICs measure the relationship between the model’s predicted accuracy and the intensity of the saliency map. A strong correlation between high-intensity areas on the saliency map and high model accuracy indicates that the method effectively emphasizes relevant image features. Meanwhile, SICs examine the link between the saliency map and the model’s class probabilities (softmax outputs). An effective saliency method should highlight areas that guide the model toward the correct classification, corresponding to the disease’s localized region in the image.

To our knowledge, this study is the first empirical investigation that uses AICs and SICs to assess saliency methods in medical image analysis using DL. This offers a solid and objective framework for determining the efficacy of saliency methods in elucidating the decision-making mechanisms of DL models for classification and detection tasks in medical imaging.

### Paper outline

The paper is organized as follows. Materials and methods section describes the materials and methods employed in this paper. Results section presents experimental results on two datasets. Conclusion section concludes and proposes future directions.

## Materials and methods

This section introduces the deep CNN models used for conducting experiments. We also detail the training process for these models and present our proposed framework, which provides an in-depth explanation of image-based saliency methods and their direct applications to DL-based models in medical image analysis.

### Datasets

We use two medical image data modalities to test the attribution framework. The choice of the two modalities depends on the availability of data. Other types of modalities are also applicable to the attribution framework. We leave this for future work.

**Fig. 1:**
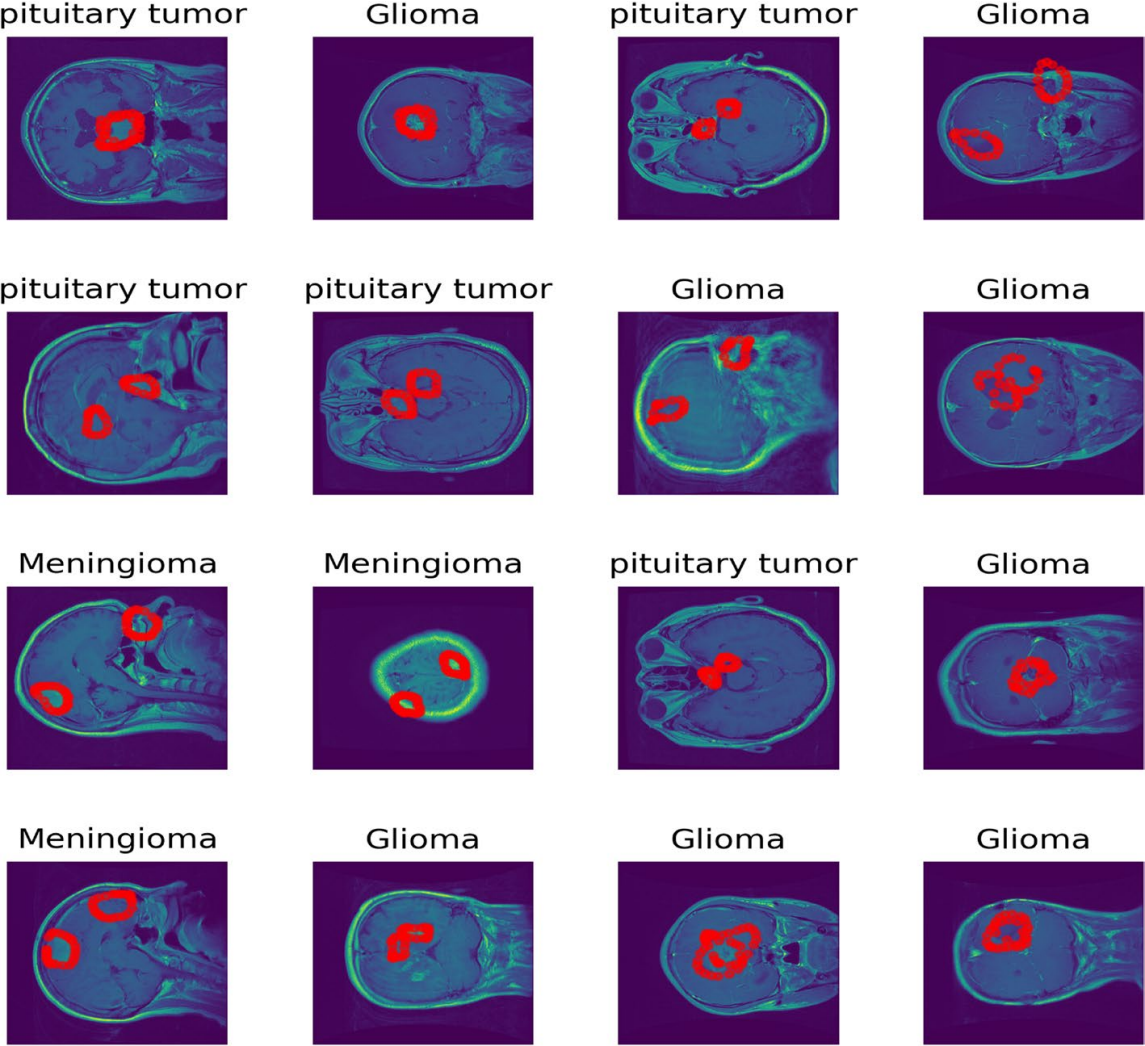
MRI Scans of Various Brain Tumors with Annotated Tumor Regions. This figure shows MRI images of different brain tumor types, with the tumor region boundaries highlighted in red. The tumor types include pituitary tumors, gliomas, and meningiomas. Each image presents a different view (axial, sagittal, or coronal) of the brain, illustrating the diversity in tumor appearance and location

**Fig. 2:**
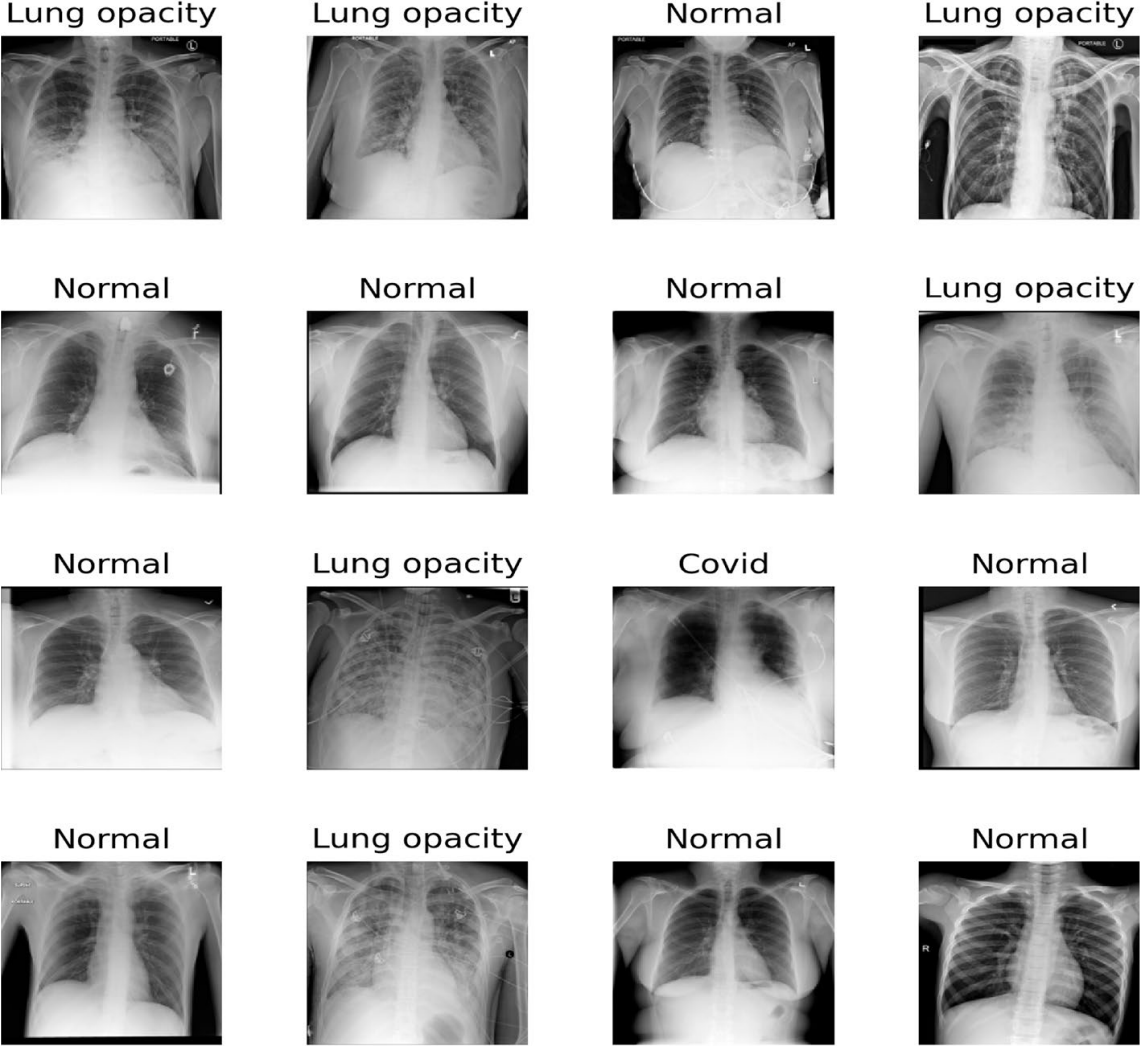
Sample chest X-ray images from the dataset used in this study, labeled with their respective conditions. The conditions include Normal, Lung opacity, and Covid. The dataset was curated from multiple international COVID-19 X-ray testing centers during several periods. The diversity in conditions showcases the varying features that the models need to identify for accurate classification

The brain tumors MRI dataset [33] is used. MRI data typically comprises a 3D tensor. However, the dataset provided in [33] is transformed from 3D tensors into 2D slices. Specifically, it includes contrast-enhanced MRI (CE-MRI) T1-weighted images, amounting to 3064 slices obtained from 233 patients. It includes 708 Meningiomas, 1426 Gliomas, and 930 Pituitary tumors. In each slice, the tumor boundary is manually delineated and verified by radiologists. We have plotted 16 random samples from the three classes with tumor borders depicted in red as shown in Fig. 1. These 2D slices of T1-weighted images train standard deep CNNs for a 3-class classification task into Glioma, Meningioma, and Pituitary tumors. The input to each model is a $\mathbb {R}^{225\times 225\times 1}$ tensor that is a resized version of the original $\mathbb {R}^{512\times 512}$ image slices primarily due to computational concerns. Unlike the brain cancer MRI dataset which comes with segmentation masks from experts in the field, the COVID-19 X-ray dataset [34] used in this work has no ground truth segmentation masks. This was chosen as an edge-case analysis because a vast majority of datasets do not have segmentation masks. This dataset was curated from multiple international COVID-19 X-ray testing facilities during several periods. The dataset is made up of an unbalanced percentage of the four classes in which we have 48.2 $\%$ normal X-ray images, 28.4 $\%$ cases with lung opacity, 17.1 $\%$ of COVID-19 patients and $6.4\%$ of patients with viral pneumonia of the 19,820 total images in the dataset. This unbalanced nature of the dataset comes with its classification challenges, which has prompted several researchers to implement DL methods to classify the dataset. Out of the four classes, for consistency with the other datasets used in this work, we choose to classify three classes (i.e., Normal, Lung Opacity, and COVID-19). For an in-depth discussion of works that deal with this dataset, we refer to [35]. Figure 2 shows 16 selected random samples. Table 1 summarizes those three datasets.

**Table 1:** The 2 datasets comprising different modalities used to carry out experiments in this study

Source	Classes	Number of samples	Total	Modality	Segmented
Brain Tumor dataset [33]	Meningioma	708	3064	MRI	✓
	Glioma	1,426			
	Pituitary tumor	930			
COVID-19 database [34]	COVID-19	3,616	19,820	X-ray	✗
	Normal	10,192			
	Lung Opacity	6,012			

### Deep learning architectures

We use 9 standard CNN architectures: Visual Geometric Group (VGG16 and VGG19 [7]), Deep Residual Network (ResNet50, ResNet50V2) [4], Densely Connected Convolutional Networks (DenseNet) [36], DL with Depthwise Separable Convolutions (Xception) [5], Going deeper with convolutions (Inception) [37], a hybrid deep Inception and ResNet and EfficientNet: Rethinking model scaling for convolutional neural networks [38] for classifying COVID-19 X-ray images and brain tumors from the T1-weighted MRI slices. The choice of these deep models is explained by the fact that they are modern techniques that are widely used in solving vision tasks and by extension medical image feature extraction for prediction.

### Image-based saliency methods and proposed framework

**Fig. 3:**
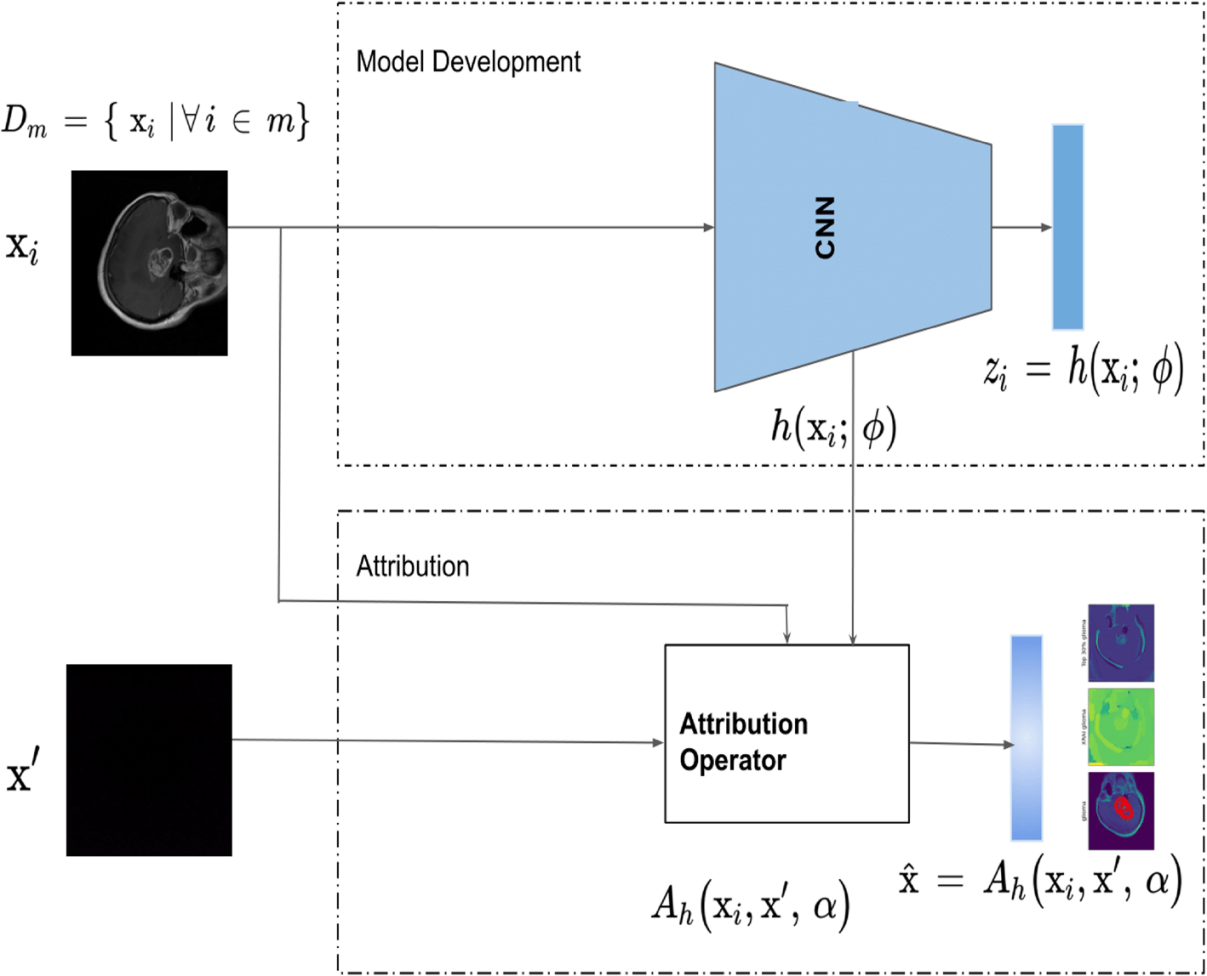
An illustration of model development and explainability pipeline for a path-based saliency method. A dataset of *m* samples say T1-weighted contrast-enhanced image slices, for example, is the input to a standard CNN classification model depicted in the figure as $h(\cdot )$ that learns the non-linear mapping of the features to the output labels. $h(\cdot )$ is utilized with an attribution operator $A_h$ to attribute salient features $\hat{\textbf{x}}$ of the input image. $A_h$ is an operator that can be used with varied different architectures. This proposed framework is general and can be applied to any problem instances where explainability is vital

To facilitate the explainability of model inference mechanisms, which is crucial for building trust in clinical applications of DL-based CAD systems, we have investigated a variety of saliency methods. These saliency methods are integrated into the proposed framework, depicted in Fig. 3. According to [39], effective attribution methods must satisfy the fundamental axioms of *Sensitivity and Implementation Invariance*. All selected saliency methods in this study adhere to these axioms.

The saliency methods evaluated include both gradient-based and gradient-free techniques. Adaptive path-based integrated gradients (APMs), which are gradient-based, are useful in reducing noise in attribution maps, which is critical for medical imaging diagnostics. Gradient-free techniques do not rely on model gradients, making them suitable for non-differentiable models or scenarios where gradients are noisy. Class Activation Mapping (CAM) and its derivatives are effective in highlighting high-level activations for visual localization, providing clear insights into decision-making processes. Each method’s distinct characteristics justify their inclusion and comparison in this study, aimed at enhancing diagnostic and patient outcomes in medical imaging.

The specific saliency methods employed in this study include several prominent techniques. Vanilla Gradient [40] computes the gradient of the output with respect to the input image, highlighting the most influential pixels for the target class prediction. Integrated Gradients (IG)[39], which are gradient-based, attribute the model’s prediction to its input features by integrating the gradients along the path from a baseline to the input image. SmoothGrad IG [41] enhances IG by averaging the gradients of multiple noisy copies of the input image, thus reducing visual noise in the saliency maps. Guided Integrated Gradient (GIG) [42] refines IG further by guiding the gradients to produce less noisy and more interpretable saliency maps. eXplanation with Ranked Area Integrals (XRAI) [43] generates region-based attributions by ranking areas based on their contribution to the prediction, providing a more holistic view of important regions. GradCAM [21] uses the gradients of the target class flowing into the final convolutional layer to produce a coarse localization map of important regions in the image. GradCAM++ [44] improves upon GradCAM by providing better localization by considering the importance of each neuron in the last convolutional layer. ScoreCAM [45], unlike gradient-based methods, uses the model’s confidence scores to weigh the importance of each activation map, potentially leading to more accurate and less noisy explanations.

These methods are integrated into the proposed framework to analyze the attribution of salient features in medical images. As shown in Fig. 3, a dataset of *m* samples is input into a standard CNN classification model. The model, represented as $h(\cdot )$, learns the non-linear mapping of features to output labels. The trained model is then utilized together with an attribution operator $A_h$, which could be any of the saliency methods, to attribute salient features $\hat{\textbf{x}}$ of the input image. This operator $A_h$ is versatile and can be applied to any problem where explainability is essential for building trust in the model’s inference mechanism.

### Quantitative and empirical assessment of saliency methods

In this work, we adapted and applied empirical methods from Kapishnikov et al. (2021) [42] for evaluating saliency frameworks in the field of medical image analysis, making slight adjustments to the image entropy calculation. Our adaptation maintained the core approach of using saliency methods to attribute importance to regions within medical images while tailoring them to meet the specific demands of medical imaging.

Our method for estimating image entropy involves computing the *Shannon entropy* of the image histogram. We begin by deriving the histogram of the original image with 256 bins and density normalization, followed by using the entropy computation as shown in Equation 1. In contrast, their method estimates image entropy by determining the file size of the image after lossless compression and calculating the buffer length as a proxy for entropy. While both approaches aim to gauge the information content of an image, ours relies on pixel intensity distribution, while theirs assesses file size post-compression.

1 $$\begin{aligned} H(X) = -\sum \limits _{i=1}^{n} p_i \log _2(p_i), \end{aligned}$$

where, *H*(*X*) represents the entropy of the image *X*, $p_i$ is the probability of occurrence of each intensity level *i* in the image histogram, and *n* is the total number of intensity levels (256 in our case).

Our approach provides a direct measure of the information content inherent in the pixel intensity distribution, capturing the relative importance of different intensity levels and offering a comprehensive understanding of the image’s complexity. In contrast, using file size post-compression as a proxy for entropy may not fully capture the nuances of the image’s content. By focusing on pixel intensity distribution, our approach offers a more intrinsic and nuanced measure of image information content, particularly crucial for tasks such as medical image analysis or pattern recognition.

This evaluation framework entails initiating the process with a completely blurred version of the medical image and incrementally reintroducing pixels identified as significant by the saliency method. We then measure the resulting image’s entropy and conduct classification tasks to correlate the model’s performance, such as accuracy, with the calculated entropy or *information level* for each medical image, resulting in Performance Information Curves (PICs). Thus, two variants of PICs were introduced – Accuracy Information Curve (AIC) and Softmax Information Curve (SIC) – to provide a more nuanced evaluation of the saliency methods’ effectiveness.

### Experimental setup

We conducted all experiments on Nvidia Quadro RTX 8000 hardware, leveraging its robust computational capabilities to handle the extensive DL training processes. For the implementation, we used the Keras API with the TensorFlow backend, enabling efficient and flexible development of the CNNs.

## Results

In this section, we present a comprehensive analysis of our experimental findings, structured around three key questions: (i) How good are these models on standard classification performance metrics? (ii) How visually explainable are studied image-based saliency-based methods? (iii) How empirically comparable are image-based saliency methods?

### How good are these models on standard classification performance metrics?

**Fig. 4:**
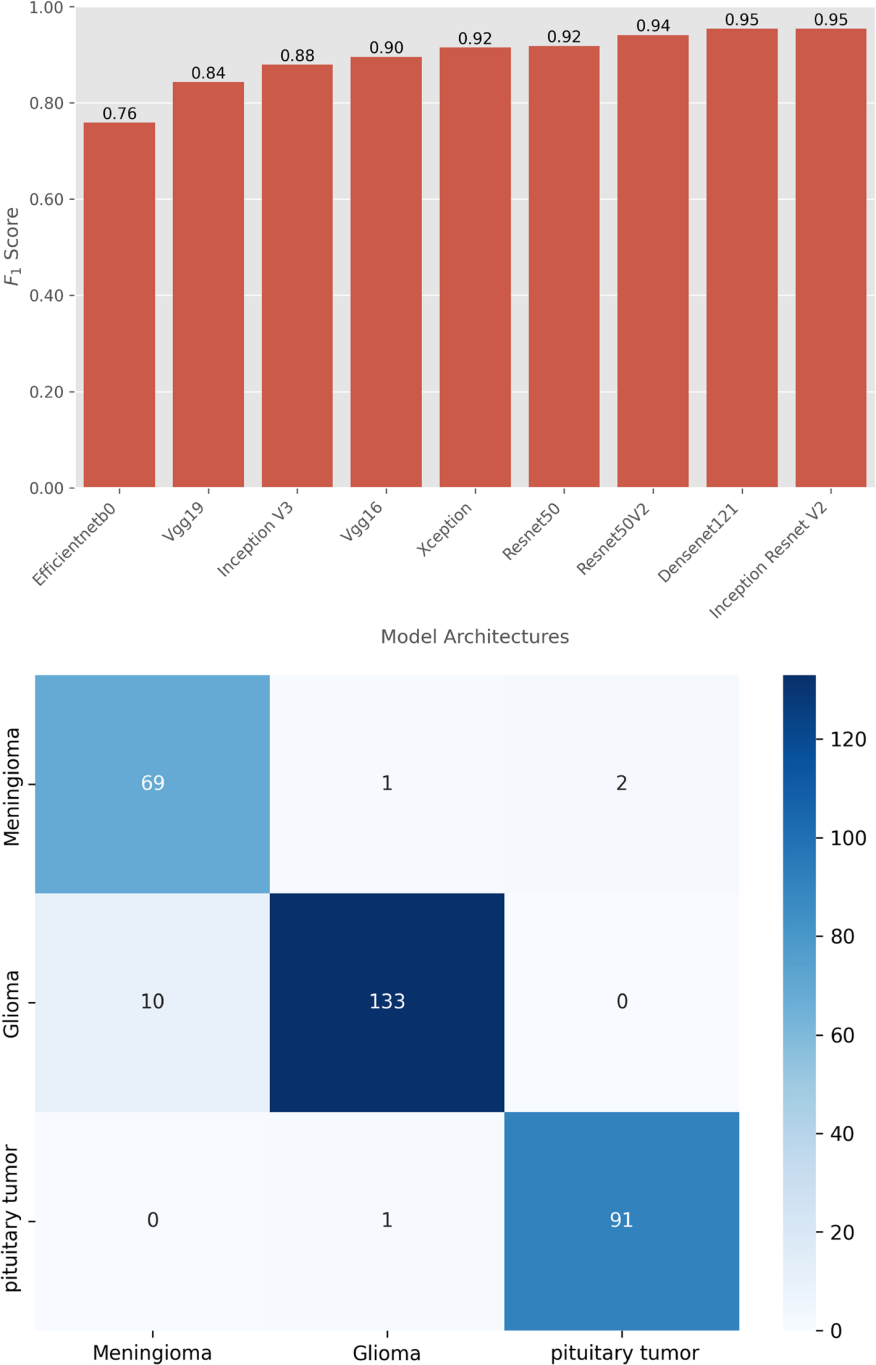
The F1 scores (top-panel) for each model are compared to assess their accuracy and robustness in classifying brain tumors into three categories: Meningioma, Glioma, and Pituitary tumor. The bottom-panel shows the confusion matrix for the top-performing model, InceptionResNetV2

**Fig. 5:**
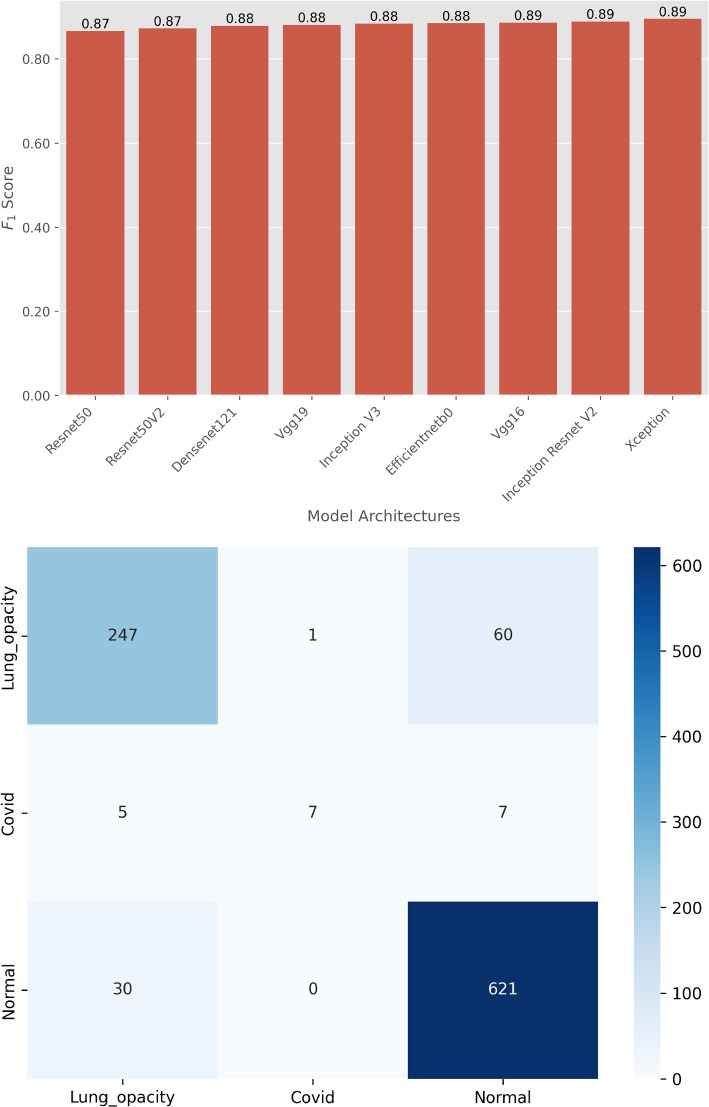
The F1 scores (top panel) for each model are compared to assess their accuracy and robustness in classifying chest X-ray images into three categories: Normal, Lung Opacity, and COVID-19. The bottom panel shows the confusion matrix for the top-performing model, Xception

We evaluated the performance of the 9 DL model architectures on classification tasks using standard metrics such as F1 score and confusion matrices as depicted in Figs. 4 and 5. Appendix 1 shows the optimal hyperparameters for training the DL models. The results provide insights into the effectiveness of each model in terms of classification accuracy and error distribution.

The performance of various DL models on brain tumor MRI classification is illustrated in Fig. 4. Figure 4 (top-panel) The bar plot presents the F1 scores of various DL model architectures evaluated on the brain MRI image testset classification task. The F1 scores for these models range from 0.76 to 0.95. The InceptionResNetV2 model achieves the highest F1 score of 0.95, indicating superior performance in accurately classifying brain tumors. EfficientNetB0, on the other hand, scores the lowest with an F1 score of 0.76, showing a relatively lower performance compared to the other models. Figure 4 (bottom-panel) shows the confusion matrix for the top-performing model, InceptionResNetV2, which displays the number of correctly and incorrectly classified cases for different types of brain tumors. The matrix shows that out of the 72 cases of Meningioma, 69 cases are correctly predicted, 1 case is misclassified as Glioma, and 2 cases are misclassified as Pituitary tumor. Out of the 143 cases of Glioma, 133 cases are correctly predicted, 10 cases are misclassified as Meningioma, and no case is misclassified as a Pituitary tumor. Out of the 92 Pituitary tumor cases, 91 cases are correctly predicted, 1 case is misclassified as Glioma, and no cases misclassified as Meningioma. This detailed breakdown demonstrates the model’s effectiveness in correctly identifying the majority of cases while highlighting specific areas where misclassifications occur, particularly in distinguishing between Meningioma and Glioma.

Figure 5 shows the performance comparison of different model architectures for COVID-19 X-ray image classification. The models were evaluated based on their ability to classify images into Normal, Lung Opacity, and COVID-19 categories. Figure 5 (top-panel) shows the F1 scores of various DL model architectures evaluated for COVID-19 classification. The F1 scores range from 0.87 to 0.89. The models perform consistently well, with minimal variation in F1 scores. Figure 5 (bottom-panel) shows the confusion matrix for the Xception model and provides a detailed view of its classification performance for chest X-ray images. The matrix shows that out of the 208 Lung opacity cases, 247 cases are correctly predicted, 1 case is misclassified as COVID-19, and 60 cases are misclassified as Normal. Out of the 19 COVID-19 cases, 7 cases are correctly predicted, 5 cases are misclassified as Lung opacity, and 7 cases are misclassified as Normal. Out of the 651 Normal cases, 621 cases are correctly predicted, no case is misclassified as COVID-19, and 30 cases are misclassified as Lung opacity. This confusion matrix highlights the Xception model’s strengths and weaknesses in COVID-19 classification. While it correctly identifies a large number of cases, there are notable misclassifications, particularly with Lung opacity being misclassified as Normal in 60 instances.

The results from the F1 scores and confusion matrices demonstrate the effectiveness of various DL architectures in medical image classification tasks. InceptionResNetV2 consistently outperforms other models in brain tumor classification, achieving the highest F1 score and demonstrating excellent accuracy. The detailed confusion matrix for InceptionResNetV2 reveals minimal misclassifications, underscoring its reliability. The performance of models on the COVID-19 X-ray dataset shows high F1 scores across different architectures, with models like Xception also performing exceptionally well. The confusion matrix for Xception indicates strong classification capabilities, although some misclassifications are present, particularly in distinguishing between Lung opacity and Normal. These results underscore the importance of selecting appropriate model architectures for specific medical image classification tasks. The high F1 scores and detailed confusion matrices provide valuable insights into each model’s strengths and areas for improvement. However, the focus of this study is not to beat SoTA performance but to provide a basis for investigating the chosen saliency methods. Therefore, the top-performing models, InceptionResNetV2 for brain tumor classification and Xception for COVID-19 classification will serve as the basis for further analysis Sections in How visually explainable are image-based saliency methods? and How empirically comparable are image-based saliency methods? sections.

### How visually explainable are image-based saliency methods?

**Fig. 6:**
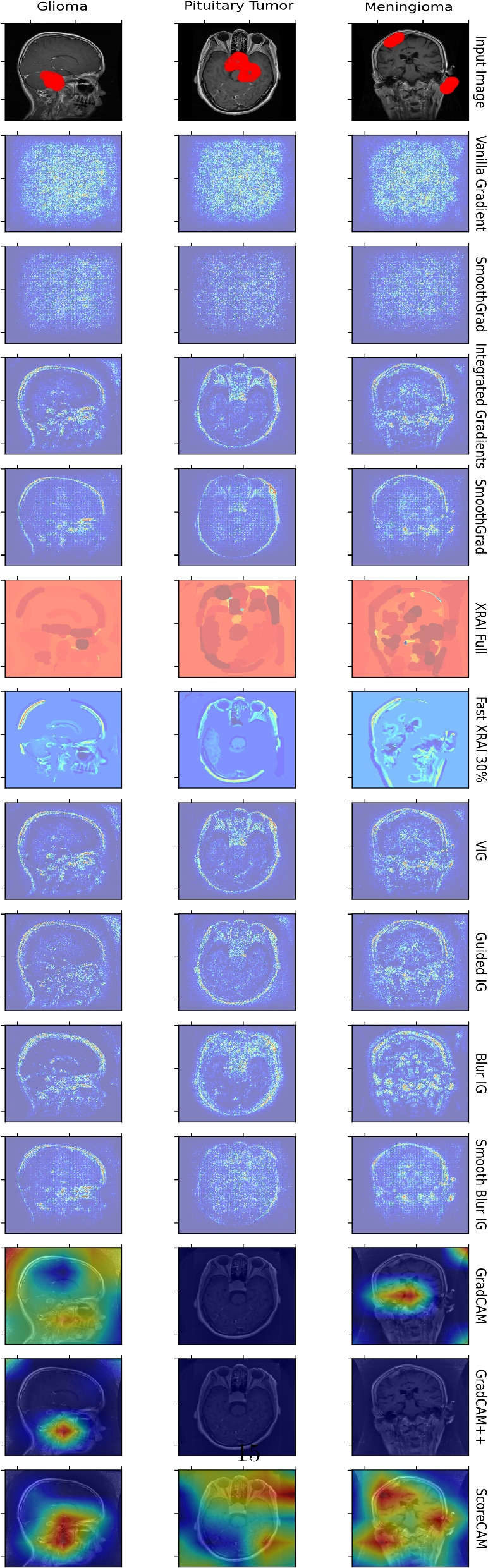
Visualization of feature attributions for brain tumor classification using various explainability methods for the best-performing model, Inception-ResNetV2. This figure displays the feature attribution maps generated by different explainability techniques for the model on three types of brain tumors: Glioma, Meningioma, and Pituitary Tumor. The columns represent the input image with ground-truth tumor boundaries followed by the attribution maps produced by each method. From visual inspection, Fast XRAI 30% and ScoreCAM outperform other methods. For Glioma, ScoreCAM effectively focuses on the tumor regions. For Meningioma, ScoreCAM highlights some tumor regions, though the heatmap shows three regions instead of the actual two. Most other methods, except GradCAM++ for Glioma, generate coarse and noisy saliency maps, particularly Vanilla Gradient and SmoothGrad. Path-integration methods tend to be more susceptible to image edges compared to GradCAM, GradCAM++, and ScoreCAM methods

Figure 6 presents the visualization of feature attributions for brain tumor classification using our proposed framework and various explainability methods applied to the Inception-ResNetV2 model. The attribution maps provide insights into the regions of the input images that significantly influence the model’s predictions for three types of brain tumors: Glioma, Meningioma, and Pituitary Tumor. The top row represents the input image with ground-truth tumor boundaries, and the other rows are attribution maps produced by each method.

From visual inspection, Fast XRAI 30% and ScoreCAM outperform other methods. For Glioma, ScoreCAM effectively focuses on the tumor regions, providing clear and accurate attributions. For Meningioma, ScoreCAM highlights some tumor regions, although the heatmap shows three regions instead of the actual two. Other methods, such as Vanilla Gradient and SmoothGrad, produce coarse and noisy saliency maps. GradCAM and GradCAM++ generate more focused heatmaps but are still less precise than ScoreCAM. Path-integration methods, like Integrated Gradients, are more susceptible to highlighting image edges rather than the tumor regions, reducing their clinical explainability.

**Fig. 7:**
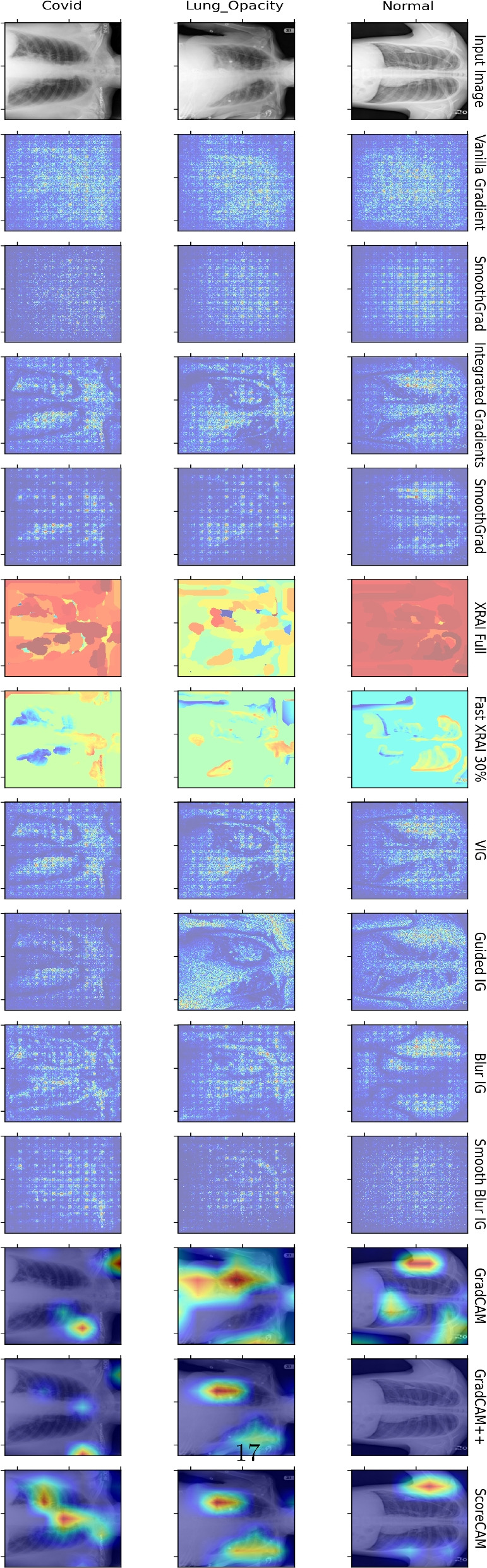
Comparison of various explainability methods applied to chest X-ray images for distinguishing between Normal, Lung Opacity, and COVID-19 cases. The figure includes the input X-ray images in the first column, followed by visualization results from different explainability methods across the subsequent columns. For each condition (Normal, Lung Opacity, and COVID-19), the visualization techniques highlight different regions of the X-ray images that contribute to the model’s decision-making process. GradCAM, GradCAM++, and ScoreCAM methods tend to produce more focused and clinically interpretable heatmaps, while other methods show more dispersed activations. XRAI and Fast XRAI provide region-based explanations that are intermediate. Unlike the brain tumor dataset, this dataset does not have ground-truth biomarkers

Figure 7 illustrates our proposed framework and application of various explainability methods on chest X-ray images for differentiating between Normal, Lung Opacity, and COVID-19 cases using the Xception model. The figure includes input X-ray images in the first row, followed by the attribution maps generated by different explainability methods. GradCAM, GradCAM++, and ScoreCAM tend to produce more focused and clinically explainable heatmaps, accurately highlighting relevant regions such as lung abnormalities. Other methods, like Vanilla Gradient and SmoothGrad, show more dispersed activations, making it challenging to interpret the model’s focus. XRAI and Fast XRAI provide region-based explanations that are intermediate, balancing between detailed local features and broader regions of interest.

The comparison of these saliency methods on the two datasets reveals the strengths and limitations of each technique in providing visual explanations. The presence of ground-truth biomarkers in the brain tumor dataset allows for a more nuanced assessment of the methods’ accuracy, whereas the COVID-19 dataset lacks such markers, relying on visual plausibility for evaluation. Overall, the findings suggest that methods like ScoreCAM, XRAI, GradCAM, and GradCAM++ offer more precise and clinically useful explanations, which are crucial for enhancing the transparency and trustworthiness of DL models in medical applications.

### How empirically comparable are image-based saliency methods?

While visual explanations provide valuable qualitative insights, it is crucial to quantitatively evaluate the effectiveness of different saliency methods. In this section, we empirically compare these methods using PICs, specifically AICs and SICs. These metrics allow us to objectively assess the correlation between the saliency map intensity and the model’s predictions, providing a comprehensive understanding of each method’s performance.

**Fig. 8:**
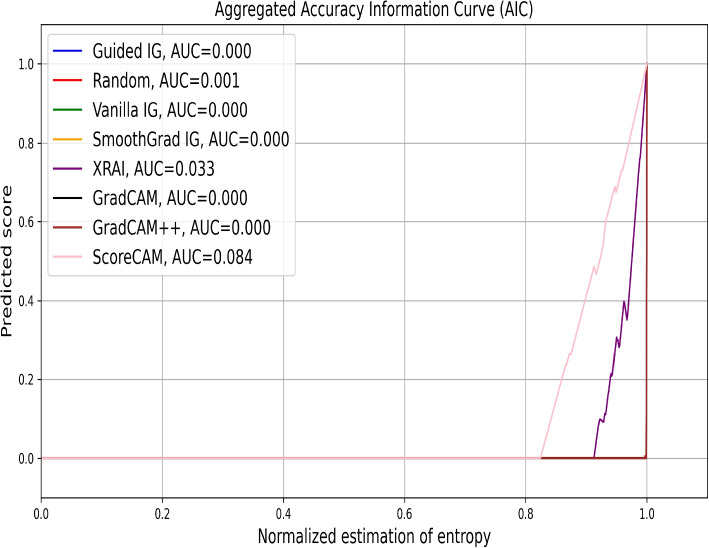
Aggregated AICs for evaluating the effectiveness of different saliency methods in attributing importance to regions of Brain Tumor MRI images for classification. The plot shows the prediction score as a function of the fraction of the image retained after reintroducing pixels identified as important by each saliency method. The area under the curve (AUC) values are provided for each method, indicating their performance in retaining critical image information necessary for accurate classification. ScoreCAM demonstrates the highest AUC of 0.084, suggesting it retains the most relevant image regions effectively, followed by XRAI with an AUC of 0.033. Other methods, including Guided IG, Vanilla IG, SmoothGrad IG, GradCAM, and GradCAM++, show minimal to zero AUC values, indicating limited effectiveness in this evaluation

In Fig. 8, we present the aggregated AICs for over 1200 data points for various saliency methods applied to brain tumor MRI classification. The AUC values indicate the effectiveness of each method in retaining critical image information necessary for accurate classification. We observe that ScoreCAM achieves the highest AUC of 0.084, followed by XRAI at 0.033. This suggests that these methods are more effective in highlighting relevant regions for the model’s predictions. In contrast, methods like Guided IG, Vanilla IG, SmoothGrad IG, GradCAM, and GradCAM++ show minimal to zero AUC values, indicating limited effectiveness. These empirical results align with our visual inspection findings, where ScoreCAM and XRAI also provided clearer and more accurate attributions.

**Fig. 9:**
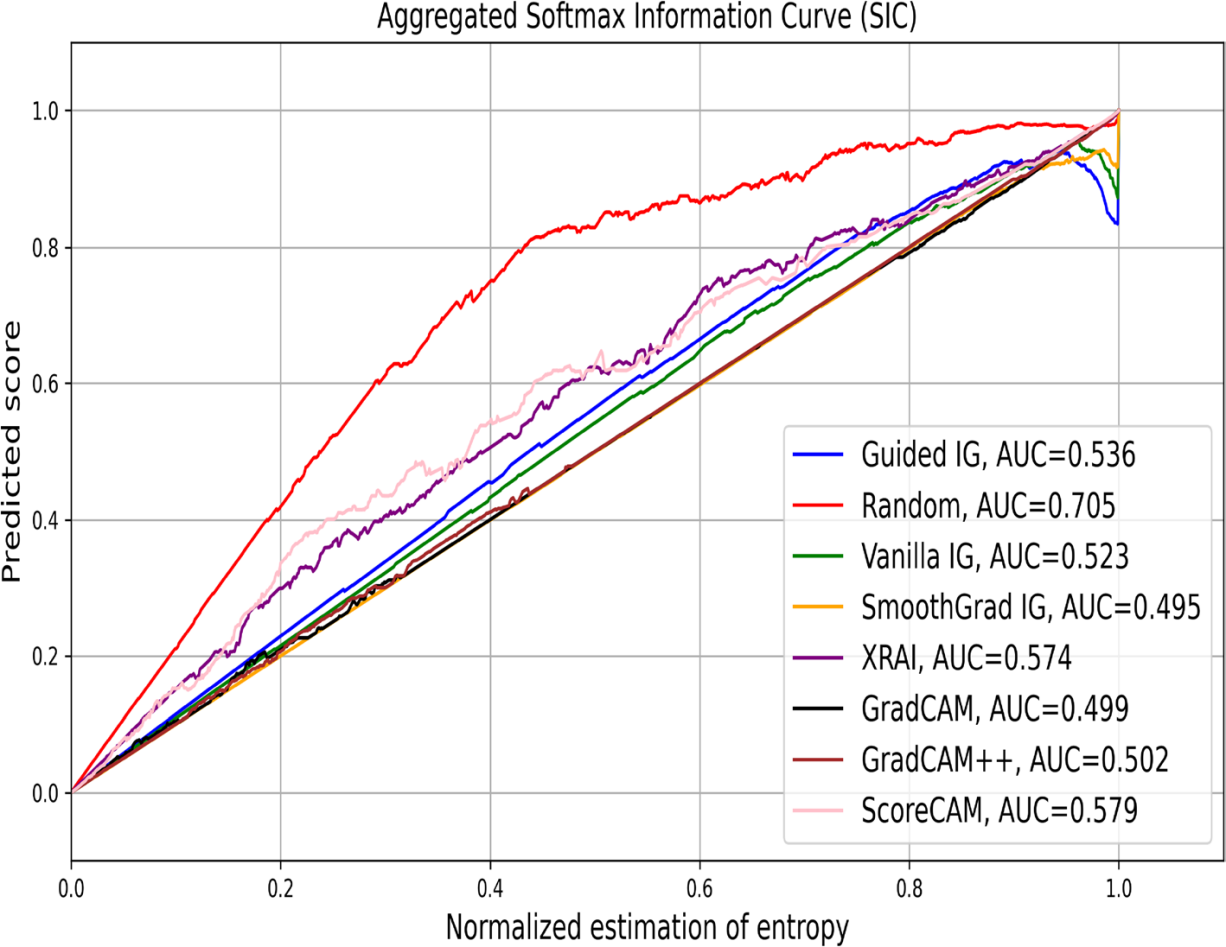
Aggregated SICs for evaluating the effectiveness of different saliency methods in attributing importance to regions of Brain Tumor MRI images. The plot shows the prediction score as a function of the fraction of the image retained after reintroducing pixels identified as significant by each saliency method. The AUC values are provided for each method, indicating their performance in retaining critical image information necessary for accurate classification. Random saliency mask, surprisingly, exhibits the highest AUC of 0.705, followed by ScoreCAM (AUC=0.579), XRAI (AUC=0.574), and Guided IG (AUC=0.536). GradCAM, GradCAM++, Vanilla IG, and SmoothGrad IG show lower AUC values, indicating less effectiveness. This analysis highlights the variability in performance among different saliency methods when applied to medical image analysis, with the Random saliency mask unexpectedly showing the highest effectiveness under this specific evaluation criterion, which indicates the instability of this metric

Figure 9 illustrates the aggregated SICs for over 1300 samples of a brain tumor MRI dataset. The SIC evaluates how well the saliency methods identify regions that contribute to the model’s class probabilities. Surprisingly, the Random saliency mask shows the highest AUC of 0.705, followed by ScoreCAM (0.579), XRAI (0.574), and Guided IG (0.536). This anomaly indicates that the Random saliency mask may retain some critical regions by chance, emphasizing the need for careful interpretation of this metric. While Guided IG and ScoreCAM perform well, their AUC values suggest that these methods provide moderately effective attributions. These findings partly contrast with our visual evaluations and AICs, where ScoreCAM was a top performer, highlighting the importance of combining visual and empirical assessments for a holistic understanding.

**Fig. 10:**
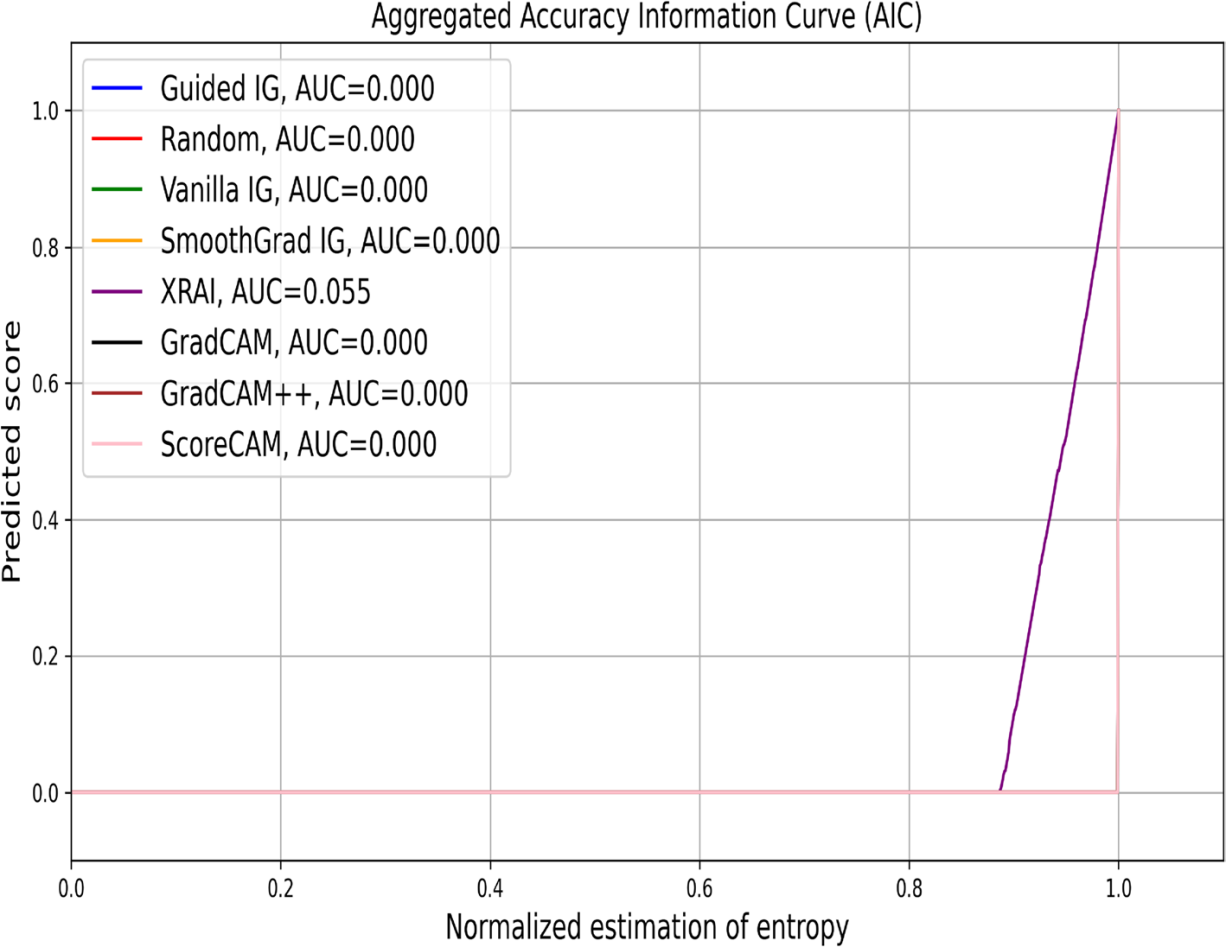
Aggregated AICs evaluating the performance of various saliency attribution methods on the chest X-ray image classification problem. The x-axis represents the fraction of the original image retained based on the saliency maps generated by each method. The y-axis shows the corresponding prediction score or accuracy. The curve for XRAI (AUC=0.055) deviates slightly from the baselines, indicating a minimal ability to identify relevant image regions for the classification task. Other methods, including ScoreCAM, Guided IG, GradCAM, and Vanilla IG, show negligible scores with an AUC of 0.000. This plot highlights the limited efficacy of these saliency techniques in attributing importance to salient regions within medical images for model explainability in this specific evaluation

In Fig. 10, we evaluate the performance of various saliency methods on chest X-ray classification tasks using the Aggregated AIC. XRAI shows a noticeable deviation from the baseline with an AUC of 0.055, indicating some effectiveness in identifying relevant regions. Other methods, including ScoreCAM, Guided IG, and Vanilla IG, closely follow the random with AUC values of 0.000, suggesting limited effectiveness in this context. This observation is consistent with our visual inspection, where methods like ScoreCAM and XRAI provided intermediate-level explanations compared to others.

**Fig. 11:**
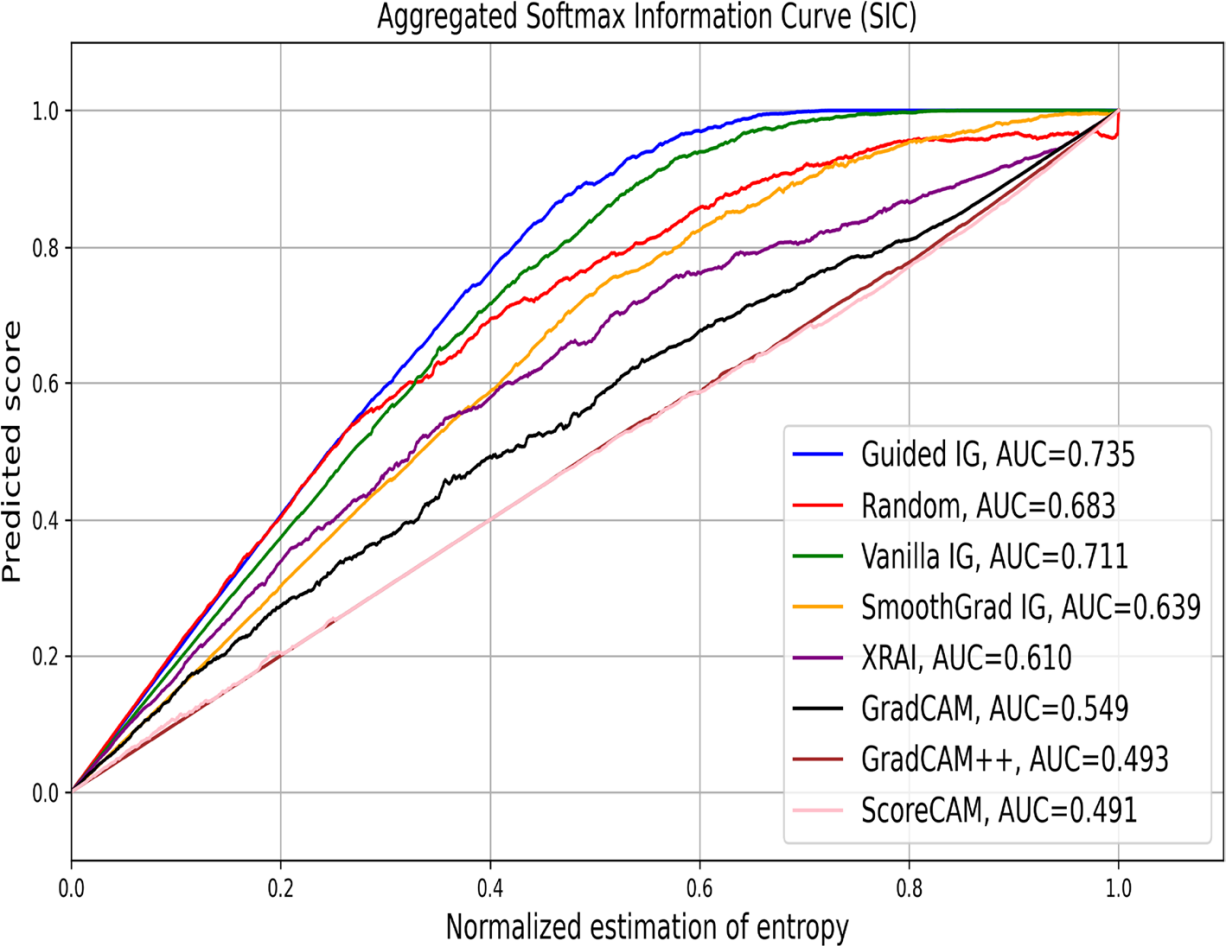
Aggregated SICs comparing the performance of various saliency methods on the chest X-ray image classification task. The x-axis represents the fraction of the image retained based on the saliency maps, and the y-axis denotes the corresponding prediction score. The guided integrated gradients (Guided IG) method achieves the highest AUC of 0.735, outperforming the random mask (AUC=0.683), vanilla integrated gradients (Vanilla IG, AUC=0.711), SmoothGrad integrated gradients (SmoothGrad IG, AUC=0.639), and other saliency methods like XRAI (AUC=0.610), GradCAM (AUC=0.594), GradCAM++ (AUC=0.493), and ScoreCAM (AUC=0.491)

Figure 11 shows the aggregated SICs for chest X-ray classification. Guided IG achieves the highest AUC of 0.735, outperforming the random mask (0.683), Vanilla IG (0.711), and SmoothGrad IG (0.639). This suggests that Guided IG is particularly effective in highlighting regions that influence the model’s class probabilities. The performance of XRAI, GradCAM, GradCAM++, and ScoreCAM is moderate, with lower AUC values (0.610, 0.594, 0.493, and 0.491 respectively), indicating less effective saliency attribution compared to Guided IG. These empirical results, similar to those for the brain tumor dataset, do not align with our visual analysis and AICs, where methods like XRAI, GradCAM, GradCAM++, and ScoreCAM provided more focused and explainable heatmaps. Thus, this metric should be cautiously used for evaluating saliency methods in given datasets.

In summary, the empirical evaluation using AICs closely aligns with the visual results. However, SICs highlight the variability in performance among different saliency methods, with instances of a random mask outperforming established saliency methods. While our visual inspections revealed clear strengths for methods like ScoreCAM and GradCAM++, the empirical metrics provide a nuanced understanding of each method’s effectiveness in retaining and highlighting relevant image regions. By combining visual and empirical analyses, we ensure a robust evaluation of saliency methods, enhancing their applicability in clinical settings.

Further analysis results are included in Appendix 2. We present a saliency analysis of the second and third-best models for each dataset. Additionally, AICs and SICs based on the entropy method from Kapishnikov et al. (2021) are provided in Appendix 2 “Buffer-size-based AICs and SICs evaluations” section. We also explore varied blurred versions of the top-performing saliency methods and their scores in Appendix 2 “Computed saliency scores for top performing models for each image-based saliency method” section.

## Conclusion

In this study, we proposed a saliency-based attribution framework and assessed various state-of-the-art saliency methods for enhancing the explainability of DL models in medical image analysis, focusing on brain tumor classification using MRI scans and COVID-19 detection using chest X-ray images. Both qualitative and quantitative evaluations provided insights into these methods’ utility in clinical settings.

Qualitative assessments showed that ScoreCAM, XRAI, GradCAM, and GradCAM++ consistently produced focused and clinically interpretable attribution maps. These methods highlighted relevant regions that aligned with known anatomical structures and disease markers, thereby enhancing model transparency and trustworthiness.

This study is the first to use AICs and SICs to quantitatively evaluate these saliency methods for medical image analysis. The AICs confirmed that ScoreCAM and XRAI effectively retained critical image information, while SICs revealed variability, with random saliency masks sometimes outperforming established methods. This underscores the need for combining qualitative and quantitative metrics for a comprehensive evaluation. Our results highlight the importance of selecting appropriate saliency methods for specific tasks. While visual explanations are valuable, empirical metrics offer a nuanced understanding of each method’s effectiveness. Combining these approaches ensures robust assessments, fostering greater trust and adoption of DL models in clinical settings.

Future research should refine empirical metrics for stability and reliability across different models and datasets, include more diverse imaging modalities, and focus on enhancing model explainability to support clinical decision-making.

## Figures and Tables

**Fig. 12:**
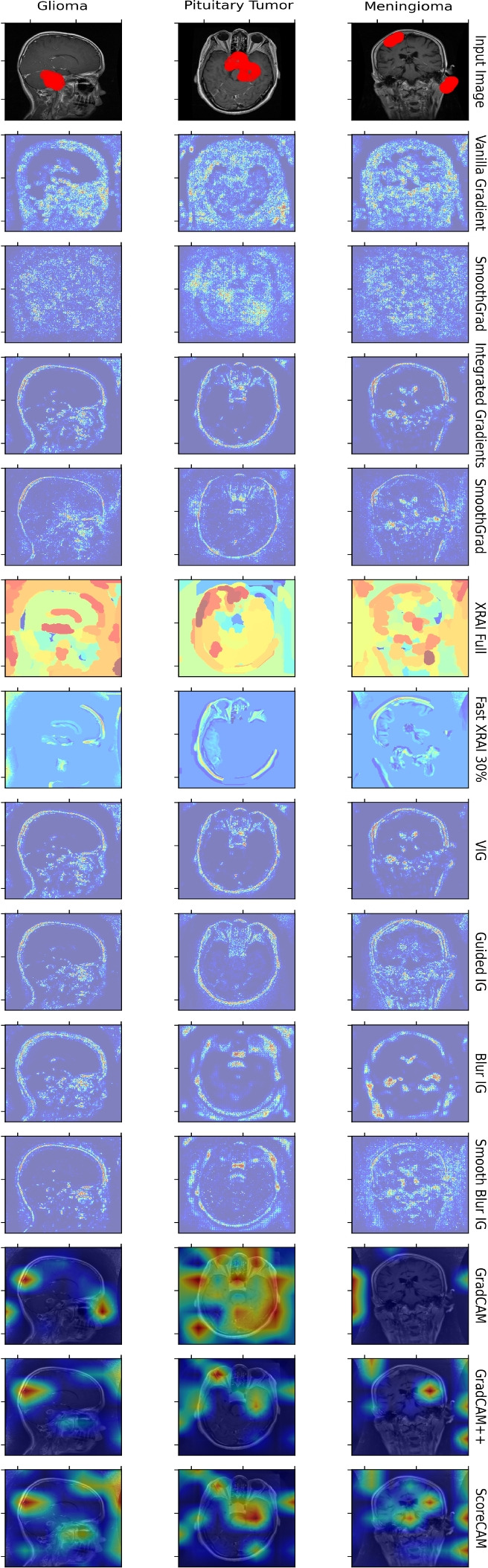
Comparative assessment of saliency techniques applied to brain MRI data using the DenseNet121 model, the second-best performing model on this dataset. Among these, ScoreCAM and GradCAM++ appear to provide the more focused highlighting of the tumor regions across all types of tumors, suggesting that they are more effective in localizing and interpreting the model’s important feature areas for accurate prediction

**Fig. 13:**
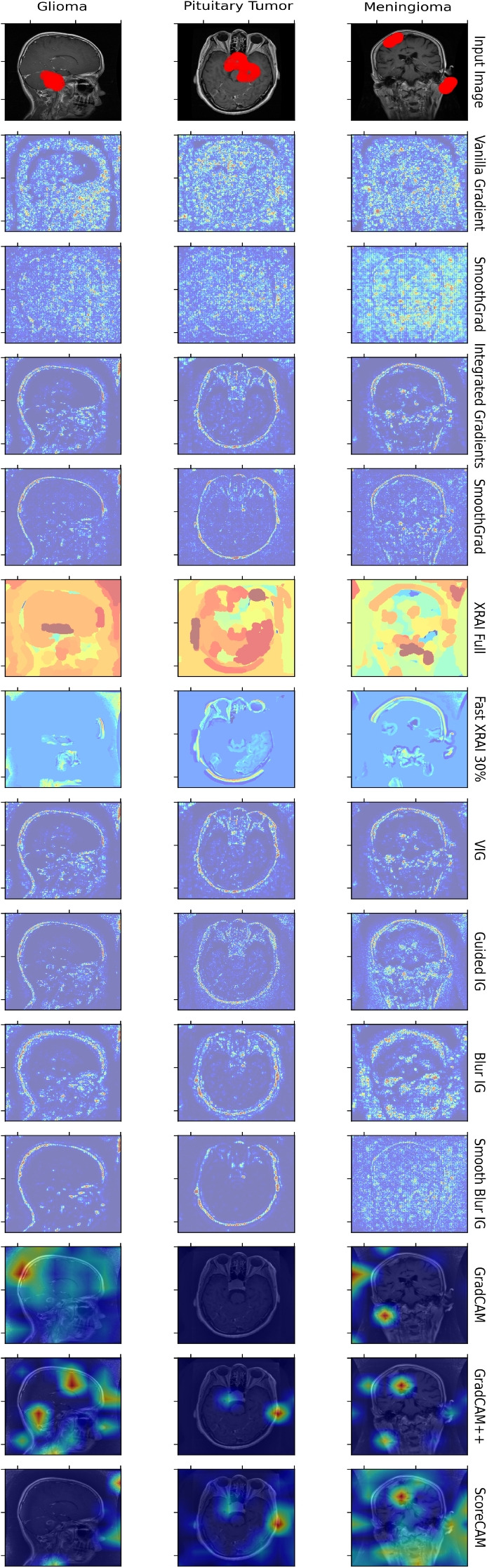
The figure presents a comparison of various saliency techniques applied to brain MRI data using a ResNetV2 model. We noticed that Fast XRAI at 30% feature masking was able to highlight relevant tumor regions across the three disease classes. Other methods produced more coarse-grained saliency masks as depicted in the plot

**Fig. 14:**
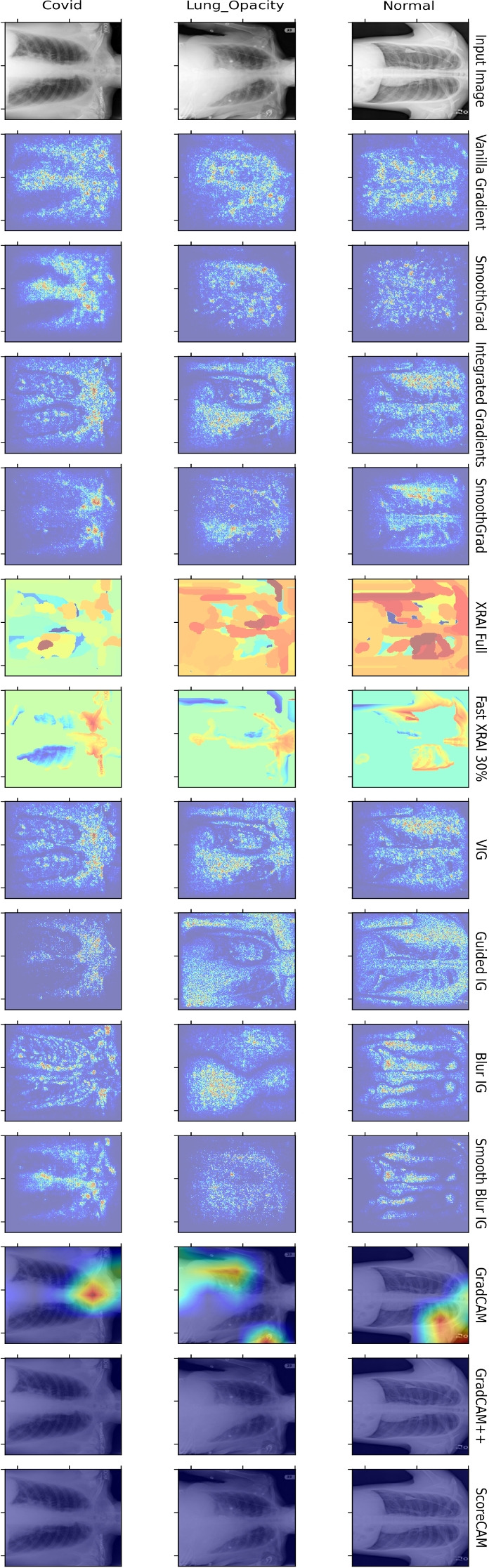
This figure illustrates a comparative evaluation of various techniques applied to chest X-ray images using an InceptionResNetV2 model, which is identified as the second-best performing model on this chest X-ray dataset. Here, we noticed that most methods other than XRAI, Fast XRAI 30%, and GradCAM did not produce clinically meaningful saliency masks contrary to the models’ prediction performance. It is, however, hard to qualitatively evaluate these methods since the dataset does not have a ground-truth segmentation mask

**Fig. 15:**
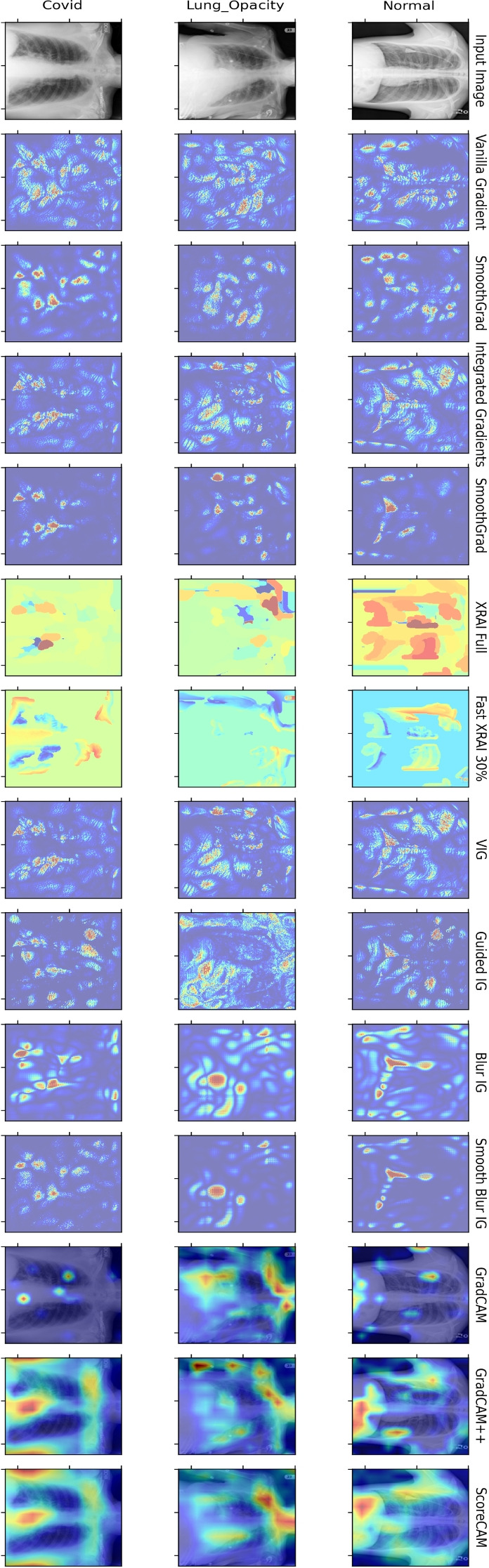
Visualization of feature importance for different chest X-ray classifications using a VGG16 model. Rows correspond to different diagnostic categories: Lung Opacity, Normal, and COVID-19. Columns represent various explainability methods. We noticed that XRAI Full, Fast XRAI 30%, GradCAM++, and ScoreCAM highlighted more meaningful features compared to other methods. It is also noticed that Fast XRAI has consistent salient features across InceptionResNetV2 and VGG16 models

**Fig. 16:**
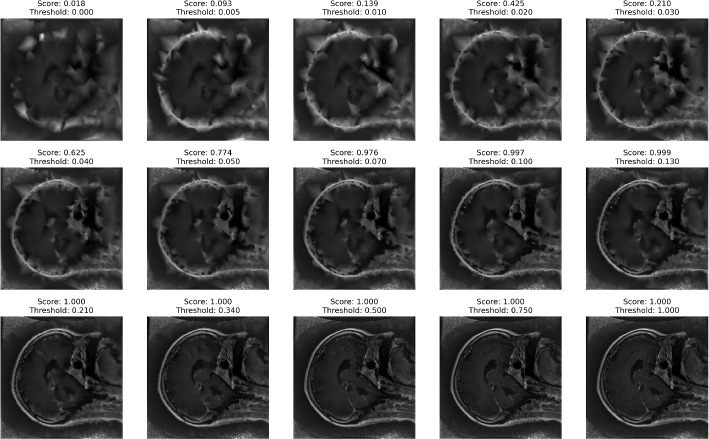
Visualization of GIG SIC scores at varying blurring thresholds for the best-performing model, Inception-ResNetV2, on the Brain Tumor dataset. Each panel displays the GIG Blurred image for a specific threshold, with the corresponding score indicating the model’s confidence level. The thresholds range from 0 to 1.0, showcasing the progression of identified significant regions as the threshold increases. Higher thresholds emphasize more critical features, aligning with the model’s high-confidence predictions, thus offering insights into the explainability and robustness of the Inception-ResNetV2 model in detecting and analyzing brain tumor regions

**Fig. 17:**
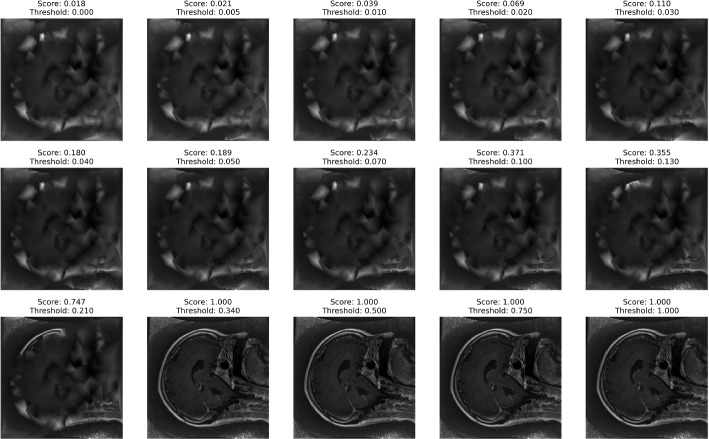
Visualization of GradCAM SIC scores at varying thresholds for the same Inception-ResNetV2, on the Brain Tumor dataset. Unlike GIG, scores only converge at higher thresholds, row three of this plot

**Fig. 18:**
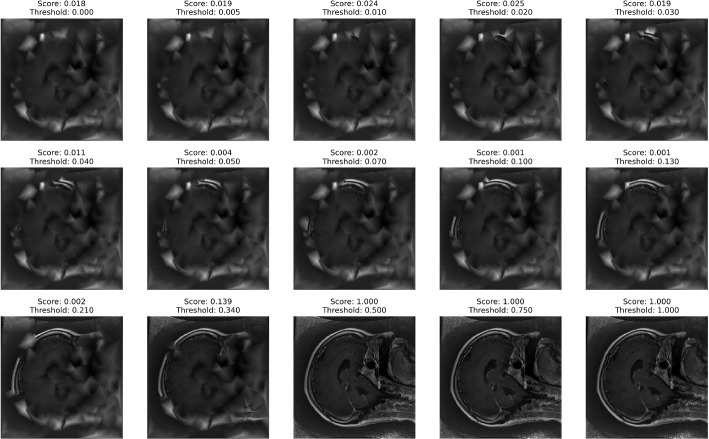
Visualization of GradCAM++ SIC scores at varying thresholds for the best-performing model, Inception-ResNetV2, on the Brain Tumor dataset. Like GradCAM, we noticed a similar trend in score convergence. However, the score converged at a threshold of 0.5 instead of 0.34 as in GradCAM

**Fig. 19:**
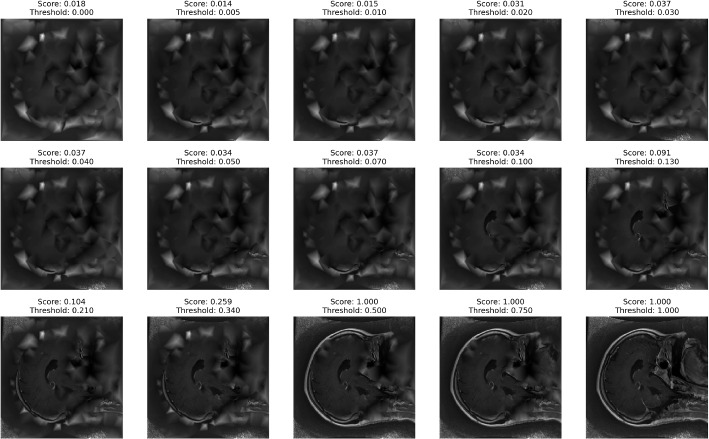
Visualization of XRAI SIC scores at varying thresholds for the best-performing model, Inception-ResNetV2, on the Brain Tumor dataset. This method also converges in the last three thresholds as depicted in the figure

**Fig. 20:**
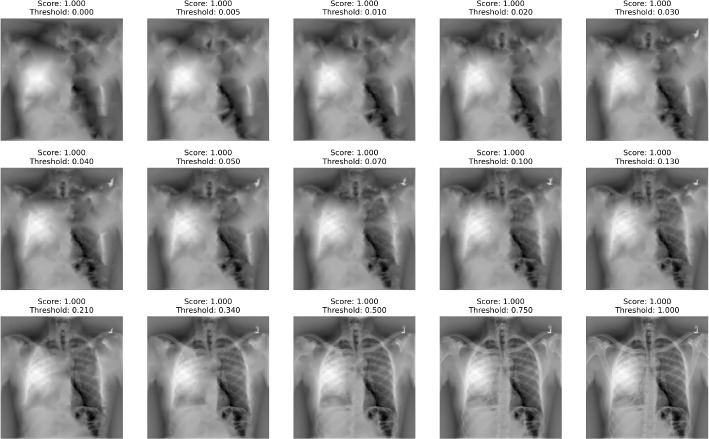
Visualization of GIG Blurred SIC scores at varying thresholds for the best-performing model, Xception, on the Chest X-ray dataset. Unlike the Brain Tumor case, we noticed a different pattern here. The scores remain constant at the different thresholds which is unexpected and counter-intuitive

**Fig. 21:**
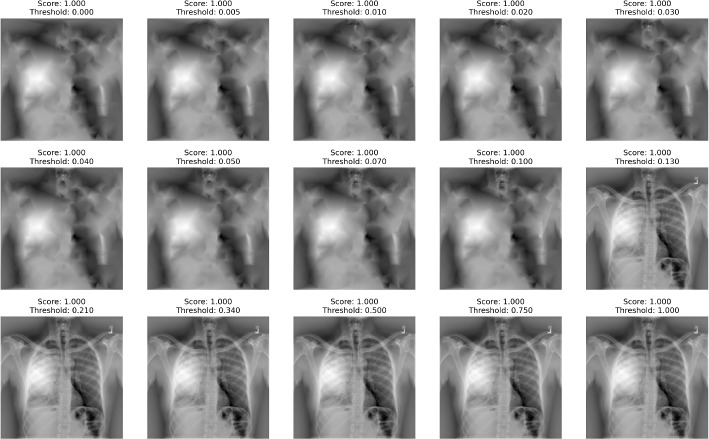
Visualization of GradCAM scores at varying thresholds for the best-performing model, Xception, on the Chest X-ray dataset. Like the previous result, we noticed a similar pattern here as the scores remain invariant across varied thresholds of blurring. This is the case for GradCAM++ and XRAI full

**Fig. 22:**
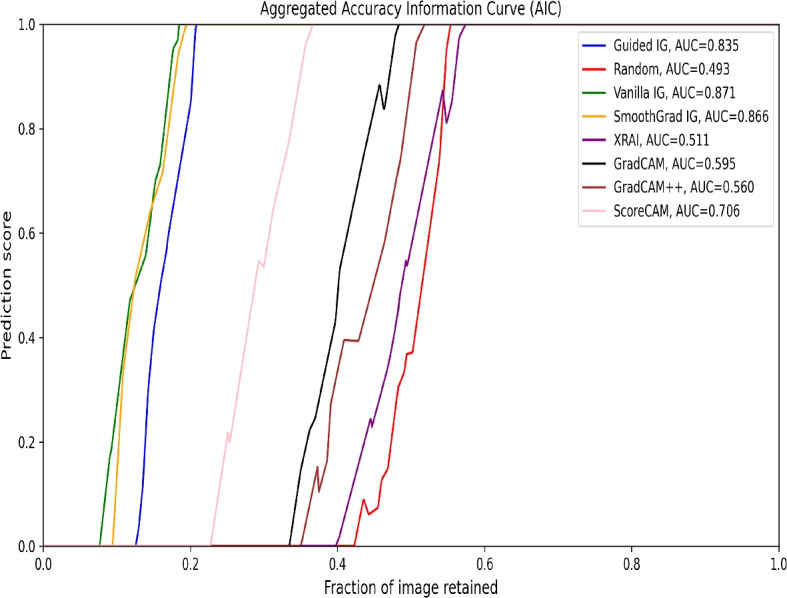
Aggregated AICs comparing the performance of various saliency methods on the Brain Tumor MRI image classification task. Vanilla IG achieves the highest AUC of 0.871, followed closely by SmoothGrad IG (0.866) and Guided IG (0.835), suggesting these methods are particularly effective in retaining relevant image regions. ScoreCAM shows a respectable AUC of 0.706, indicating good performance as well. GradCAM and GradCAM++ display moderate effectiveness with AUC values of 0.595 and 0.560, respectively. XRAI has an AUC of 0.511, and the Random saliency mask shows an AUC of 0.493, suggesting that some important regions might be retained by chance. This comparison highlights the variability of the entropy estimation to compute the saliency metric scores across datasets. This is primarily because the AUCs are not in agreement with the visual saliency results nor the Shannon entropy-based approach

**Fig. 23:**
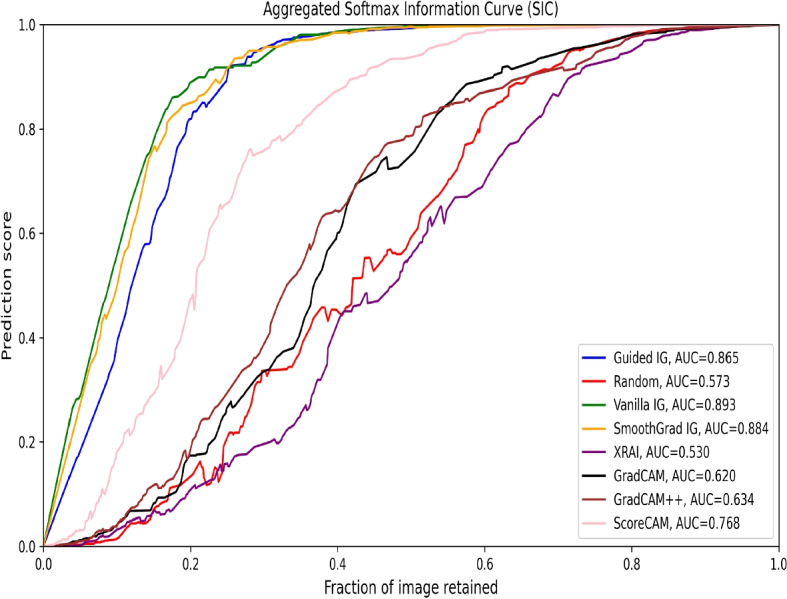
Aggregated SICs comparing the performance of various saliency methods on the Brain Tumor MRI image classification task. Vanilla IG achieves the highest AUC of 0.893, closely followed by SmoothGrad IG (0.884) and Guided IG (0.865), suggesting these methods are particularly effective in highlighting regions that influence the model’s class probabilities. ScoreCAM also performs well with an AUC of 0.768. GradCAM++ and GradCAM show moderate performance with AUC values of 0.634 and 0.620, respectively. XRAI shows an AUC of 0.530, and the Random saliency mask exhibits an AUC of 0.573, indicating some critical regions might be retained by chance. This comparison highlights the variability in this evaluation metric irrespective of the underlying approach to estimating image entropy

**Fig. 24:**
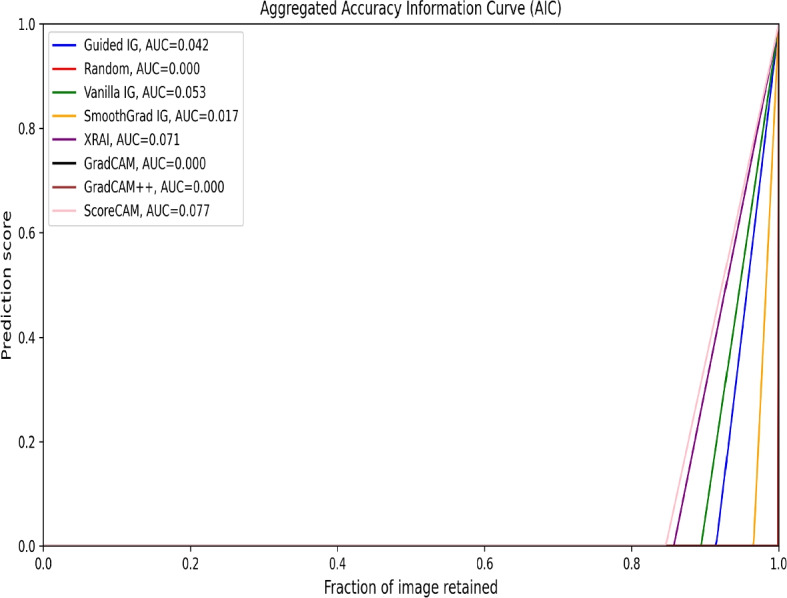
Aggregated AICs evaluating the performance of various saliency attribution methods on the Chest X-ray image classification task. ScoreCAM demonstrates the highest AUC of 0.077, suggesting it retains the most relevant image regions effectively. This is followed by XRAI with an AUC of 0.071, Vanilla IG with an AUC of 0.053, and Guided IG with an AUC of 0.042. Methods like SmoothGrad IG, GradCAM, and GradCAM++ show minimal to zero AUC values, indicating limited effectiveness in this evaluation. The overall trend highlights that some methods, particularly ScoreCAM and XRAI, provide better retention of relevant regions compared to others. This result is in line with the Shannon entropy-based approach

**Fig. 25:**
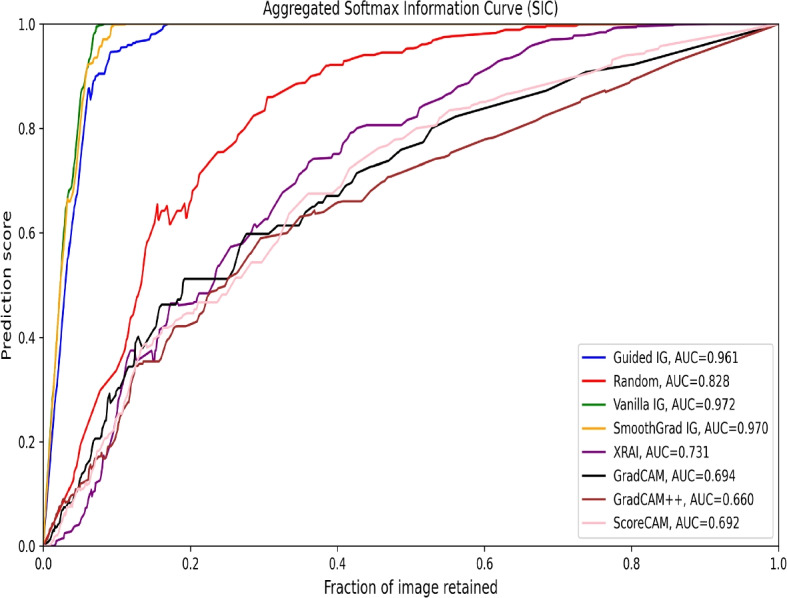
Aggregated SICs comparing the performance of various saliency methods on the Chest X-ray images. The overall trend shows that Vanilla IG achieves the highest AUC of 0.972, closely followed by SmoothGrad IG (0.970) and Guided IG (0.961). Random saliency exhibits a high AUC of 0.828, suggesting that some important regions might be retained by chance. Other methods, including XRAI (0.731), GradCAM (0.694), ScoreCAM (0.692), and GradCAM++ (0.660), show moderate performance. This detailed comparison highlights a somewhat inverse relation with the visual explainability results

## Data Availability

This research used the brain tumor dataset from the School of Biomedical Engineering Southern Medical University, Guangzhou, contains 3064 T1-weighted contrast-enhanced images with three kinds of brain tumors. The data is publicly available at Brain Tumor Dataset. The Chest X-ray dataset is publicly available at: Chest X-Ray Images (Pneumonia) Dataset.

## Appendix 1

### Models’ configuration

Table 2 shows the optimal hyperparameters for training the DL models discussed in this paper.

## Appendix 2

### Exaplanability results

**Table 2:** Training hyperparameters

Hyperparameter	Setting
Learning rate	1e-3
Batch size	64
Number of epochs	20 for Brain Tumor MRI and 40 for COVID-19 datasets
Training set	0.8
Validation set	0.1
Test set	0.1
Input shape	$\mathbb {R}^{225 \times 225 \times 1}$
Momentum	9.39e-1
Decay	3e-4
Optimizer	Stochastic Gradient Descent with Momentum (SDGM)

## Code availability

The code is available at XAIBiomedical for reproducibility.
